# Auxetic structures used in kinesiology tapes can improve form-fitting and personalization

**DOI:** 10.1038/s41598-022-17688-w

**Published:** 2022-08-05

**Authors:** Luna Meeusen, Sara Candidori, Laura Loredana Micoli, Gabriele Guidi, Tino Stanković, Serena Graziosi

**Affiliations:** 1 Engineering Design and Computing Laboratory, Department of Mechanical and Process Engineering, ETH Zürich, Tannenstrasse 3, 8092 Zürich, Switzerland; 2 Department of Mechanical Engineering, Politecnico Di Milano, Via La Masa 1, 20156 Milan, Italy; 3 Luddy School of Informatics, Computing and Engineering, Luddy Center for Artificial Intelligence, Indiana University, 1015 E 11th St, Bloomington, 47408 USA

**Keywords:** Biomedical engineering, Mechanical engineering

## Abstract

Each year 65% of young athletes and 25% of physically active adults suffer from at least one musculoskeletal injury that prevents them from continuing with physical activity, negatively influencing their physical and mental well-being. The treatment of musculoskeletal injuries with the adhesive elastic kinesiology tape (KT) decreases the recovery time. Patients can thus recommence physical exercise earlier. Here, a novel KT based on auxetic structures is proposed to simplify the application procedure and allow personalization. This novel KT exploits the form-fitting property of auxetics as well as their ability to simultaneously expand in two perpendicular directions when stretched. The auxetic contribution is tuned by optimizing the structure design using analytical equations and experimental measurements. A reentrant honeycomb topology is selected to demonstrate the validity of the proposed approach. Prototypes of auxetic KT to treat general elbow pains and muscle tenseness in the forearm are developed.

## Introduction

An injury prevents professional athletes and amateurs alike from their regular sports activities, thus negatively altering their quality of life and habits. Studies show that 65% of young athletes suffer from sports-related injuries each year^1^, and that among physically active adults, 25% of them suffered from at least one musculoskeletal injury over a one-year period^2^. It is also reported that 75% of the men and 68% of the women were unable to continue exercising due to the injury^2^. Such interruptions can negatively affect both the physical and mental status of an athlete^1^, involving fitness decrease^3^ and lowered self-esteem^4^. The latter is especially the case for athletes with a strong athletic identity^5^, for which isolation from their team members in combination with reduced performance can worsen their mental well-being^1^ and consequently even lead to depression^6^.

**Figure 1:**
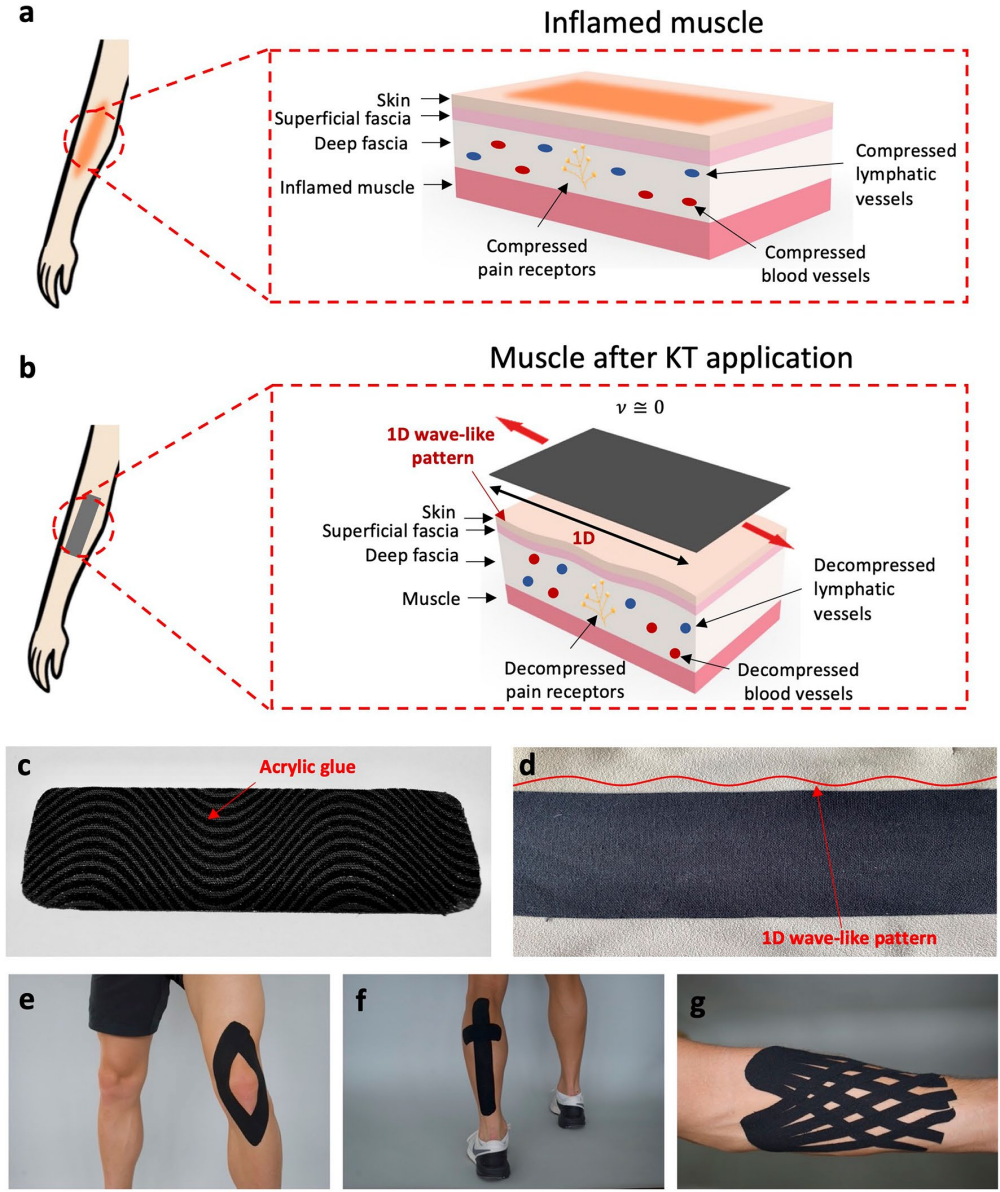
Existing KT (**a**) The compression on the skin, fascia, and muscle due to an injury. Image inspired by^22–24^. (**b**) The positive decompressive effect generated by the KT application. A one-dimensional wave-like pattern is generated on the skin. Image inspired by^22–24^. (**c**) The wave-like pattern of the acrylic adhesive at the bottom of a KT. (**d**) The one-dimensional wave-like pattern visible to the bare eye when a KT is applied to a leather sample used to mimic the skin. (**e, f, g**) Examples of KT applications on the knee (**e**) and on the calf (**f**). In (**g**) the application of a fan-shaped tape on the forearm for lymphatic drainage.

To speed up the recovery process, a combination of therapies and exercises is applied^7,8^. Nowadays, the recovery most often involves an adjunctive treatment in which a flexible and adhesive tape, called kinesiology tape (KT), is used. Invented in the 1970s by Kenzo Kase and internationalized by Japanese volleyball players during the Seoul 1988 Summer Olympics^9^, in its current design, the KT is realized as a cotton fabric wrapped around elastic polymer fibers^10^. It allows patients to recommence their training at an earlier stage compared, for example, to the standard inelastic tape, as it does not restrict movements^7,8^. The tape is applied to the athlete/patient’s skin at the location of the injured muscles, ligaments, or joints, which causes the compression of the surrounding blood and lymphatic vessels, as well as the pain receptors (Fig. 1a), to ultimately increase pain and produce swelling^11^. However, the application of the KT lifts the skin around the inflammation, separating it from the tissue below and thus reducing the compression on blood and lymphatic vessels as well as the pain receptors (Fig. 1b)^12,13^. This effect is propagated to the injured muscle, joint or ligament, and surrounding tissue to facilitate the natural healing process^9^ and post-injury treatment^14,15^, enhance muscle activity^16,17^ and pain modulation^7,8,14^, provide neuro-sensory feedback to improve proprioception^7,18^ and assist in injury prevention^18,19^. The KT adhesion at the injury location occurs due to an acrylic glue^7,10,20^, which is applied underneath the tape in a wave-like pattern (Fig. 1c) for strong attachment^21^. The beneficial effect of the KT arises because the tape is first pre-stretched on average by a 25–75% extension and then applied to the skin^10,20^, which, as a result, folds up in a wave-like pattern (Fig. 1d).

Hence, the elastic behavior of the tape, characterized by a close to zero Poisson's ratio in combination with strong adhesion between the tape and the skin, creates an effect that recoils and folds up the skin in a longitudinal wave-like manner (Fig. 1b, d). However, since the weaving pattern of the fabric allows for the tape to expand only axially upon tension, the effect on the skin is one-dimensional, with no change in size in the transverse direction (Fig. 1d). Despite the inconclusive supporting evidence regarding the therapeutic efficacy of the KT^7,13,25,26^ for various treatments, it is still observed as an indispensable therapy amongst athletes and recreationists alike^21^.

The tape is usually applied by professionals or even patients themselves^7,8^. Due to its elastic behavior, in the majority of the cases, the tape is only applied along the length of the muscles, and the stimulus on the muscle is, as already underlined, one-dimensional^27^. Therefore, to treat larger affected areas, several pieces of KT are required (Fig. 1e, f, g)^16^. The application procedure thus comprises several manual steps involving cutting the KT, into multiple pieces, in combination with directional strategy applications to achieve the desired effect^7,16^. For example, to treat muscle tenseness in the forearm^28^ several pieces of tape are needed at the area of injury. In some instances, the pieces need to be cut to specific shapes making the application procedure complex, as in the case of fan-shaped strips to treat swellings (Fig. 1g)^21^. It is also worth underlying that the literature^7^ provides little or no evidence on how training and education of practitioners relate to treatment outcomes, especially in the case of more complex taping procedures.

**Figure 2:**
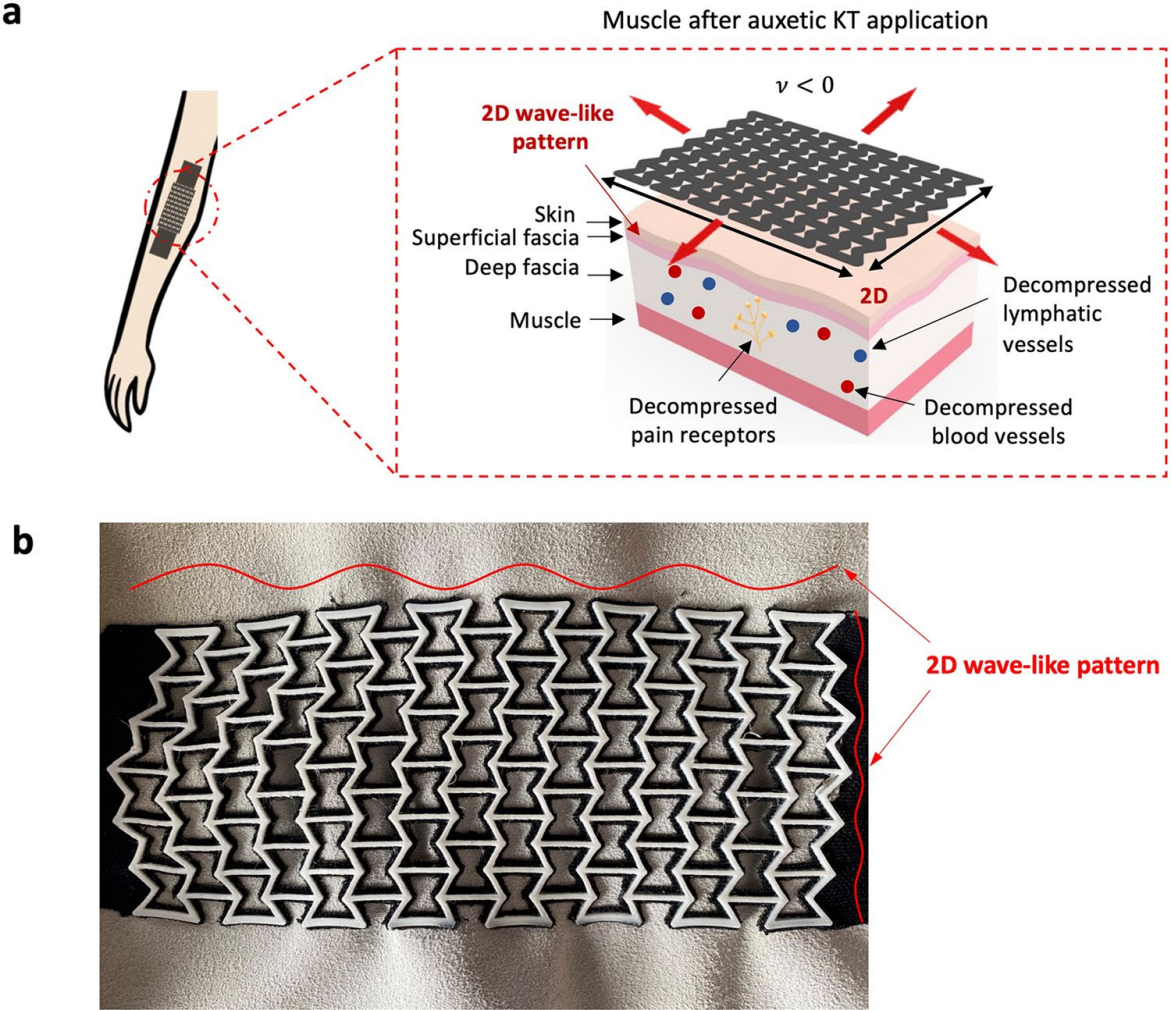
Novel auxetic KT (**a**) The positive decompressive effect generated by the auxetic KT application. A two-dimensional wave-like pattern is generated on the skin. Image inspired by^22–24^. (**b**) The two-dimensional wave-like pattern visible to the bare eye when a KT is applied on a leather sample used to mimic the skin.

Here, to simplify the application procedure of the tape to only one piece at the area of injury, instead of several, and to facilitate personalization, we propose a novel KT design based on auxetic structures. Auxetic structures are characterized by a negative Poisson's ratio $v$ which enables them to expand simultaneously in two perpendicular directions when a tension load is applied^29,30^. As such, auxetic structures found extensive applications in the development of textile-based smart wound dressings and bandages^31,32^. The structures are also well-known for their form-fitting properties, allowed by their capability to generate a synclastic curvature because of an out-of-plane bending^29,33^. The benefits of solutions capable of properly adhering to the body are privileged in the biomedical field. Indeed, for example, first 3D-printed fabrics reinforced with silicone are used to increase the efficacy of hypertrophic scar treatments^34^. Then, the beneficial effects of auxetic structures’ synclastic property, combined with a compressive bandage, are explored for the same objective^35^ in creating a stable level of pressure, during body motions, over the treated area^34^. When integrating an auxetic structure into a KT, the otherwise one-dimensional wave-like pattern created on the skin (Fig. 1b, d) is transformed into a two-dimensional one (Fig. 2). This can simplify the application procedure either for the physiotherapist or the end-user because it reduces the number of strips with complex directional strategies to be applied to achieve the positive decompressive effect (Fig. 1e, f, g), especially in cases of larger affected areas. The formfitting capability of the auxetic pattern is advantageous in terms of wearability and adhesion by reducing the risk of KT detaching because it allows the tape to properly adhere to curved body parts (e.g., joints), such as a knee or an elbow, both of which are parts prone to injures.

A simplified procedure would lead to a more frequent application of the KT, which reduces the patient's healing time: the simpler the application procedure is, the more motivated the patients are to apply the tape more often. Finally, the auxetic KT can be personalized for each patient by tuning the parameters of the auxetic design to change its mechanical behavior (i.e., the structure stiffness) and, consequently, the level of the decompressive effect it exerts on the injured area. The auxetic KT can be used to improve the treatment of the following injuries as it shows advantages over the taping methods with the existing KT: blood effusions, swellings, lymphatic blockages (in these three cases, the two-dimensional effect can be used to replace the complex fan-shaped taping, Fig. 1g); general pain at joints or muscles; pain points within muscles (the two-dimensional effect can replace the two tapes placed over the tension point); malpositioned muscles or joints (incorrect posture), fascia adhesion (the tape can cover a larger area and its effect is in multiple directions); scared tissue (with the auxetic KT only one piece is necessary instead of numerous to stimulate the skin). These additional benefits over the existing KT are likely to be well perceived by the physiotherapists and end-users of the tape as the market is rapidly growing, and there is a high demand for the product.

The reentrant honeycomb topology is selected to demonstrate the potential of the novel KT design. We started from this topology because in the literature its behavior has been extensively studied and formalized analytically with a focus on its main geometrical/design parameters. To prototype the design, first, an auxetic structure is fabricated by laser cutting an existing KT. Afterward, an additional auxetic structure is 3D-printed on the laser cut tape using thermoplastic polyurethane (TPU) material and fused filament fabrication (FFF) technology. In the first part of our work, analytical equations and experimental tests are used to determine the unit-cell geometry for the novel KT design based on the mechanical performance of five different reentrant honeycombs. In the second part, the behavior of an auxetic KT is experimentally compared to a standard KT. Prototypes are developed for two different types of injuries. One prototype is conceived to treat general elbow pain, while the other, muscle tenseness in the forearm.

## Results

### Tuning of the auxetic structure design

**Figure 3:**
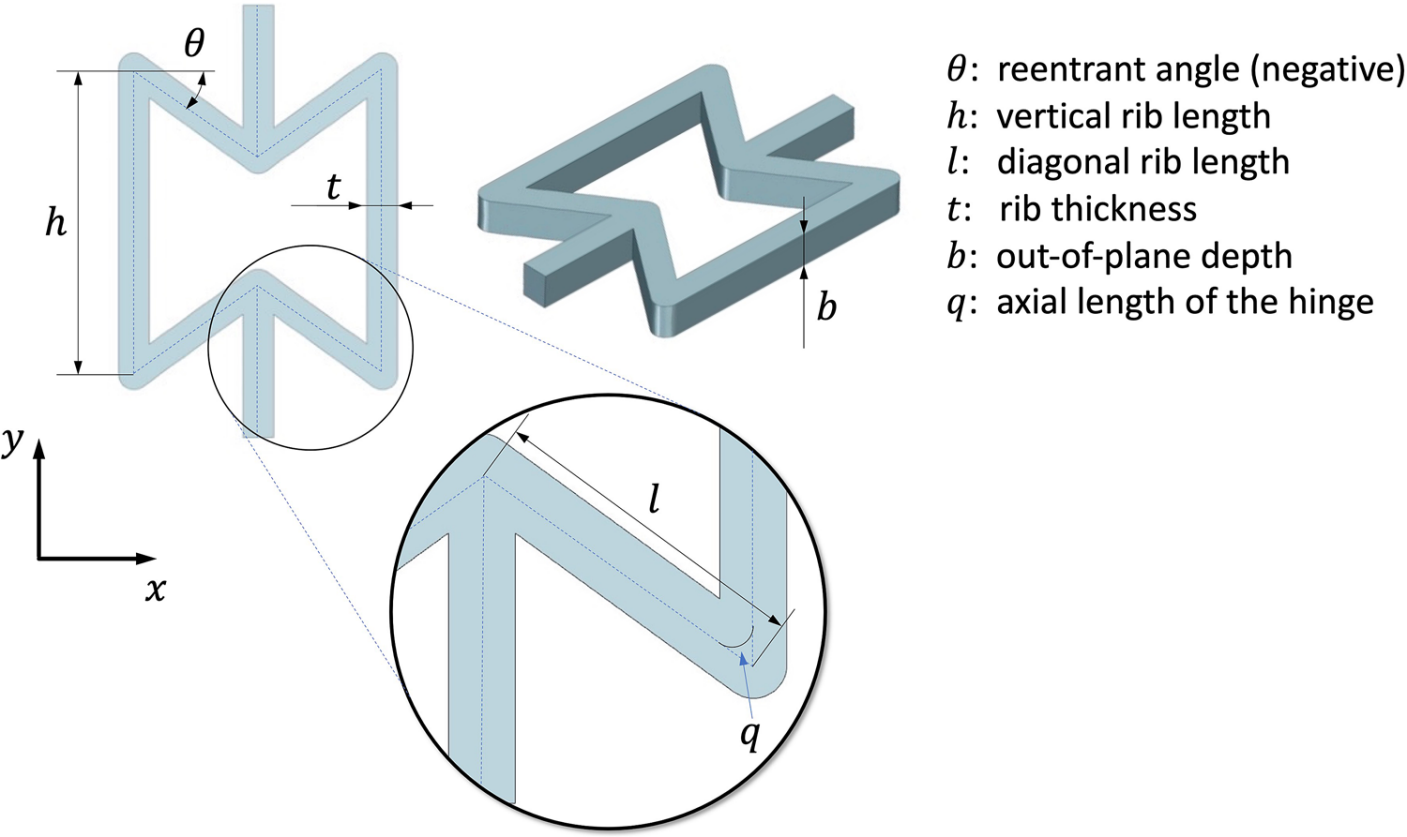
The reentrant honeycomb design and its main geometrical parameters.

When a tensional force acts on a reentrant honeycomb, three different deformation mechanisms operate on the unit cell: hinging (hg) at the cell nodes, flexing (f), and stretching (s) of the cell beams^36–38^. Combining these three effects, the Poisson's ratio of the structure can be analytically calculated as [Eq. (1)]^39–41^:

1 $$v = - \sin (\theta )\left( {h/l + {\text{sin}}\left( \theta \right)} \right)\left[ {\frac{{ - \frac{1}{{K_{{\text{f}}} }} - \frac{1}{{K_{{{\text{hg}}}} }} + \frac{1}{{K_{{\text{s}}} }}}}{{\frac{{{\text{cos}}^{2} \left( \theta \right)}}{{K_{{\text{f}}} }} + \frac{{{\text{cos}}^{2} \left( \theta \right)}}{{K_{{{\text{hg}}}} }} + \frac{{2h/l + {\text{sin}}^{2} \left( \theta \right)}}{{K_{{\text{s}}} }}}}} \right]$$

where $\theta$, $l$, $h$, $b$, $t$ are geometrical parameters (Fig. 3) while $K_{{\text{f}}}$, $K_{{{\text{hg}}}}$, and $K_{{\text{s}}}$ [Eqs. (2)–(5)] are force constants that depend on the material properties (Young's modulus $E_{{\text{s}}}$ and Poisson's ratio $v_{{\text{s}}}$ of the material).

The equations of the force constants are^39–41^:

2 $$K_{{{\text{hg}}_{{{\text{global}}}} }} = \frac{{G_{{\text{s}}} bt}}{l}$$

3 $$K_{{{\text{hg}}_{{{\text{local}}}} }} = \frac{{E_{{\text{s}}} bt^{3} }}{{6l^{2} q}}$$

4 $$K_{{\text{f}}} = \frac{{E_{{\text{s}}} bt^{3} }}{{l^{3} }}$$

5 $$K_{{\text{s}}} = \frac{{E_{{\text{s}}} bt}}{l}$$

where $q$ is the axial length of the hinge (Fig. 3) and $G_{{\text{s}}}$ is the shear modulus, which can be derived from the Young's modulus and the Poisson's ratio of the material as [Eq. (6)]:

6 $$G_{{\text{s}}} = \frac{{E_{{\text{s}}} }}{{2\left( {1 + v_{{\text{s}}} } \right)}}$$

The hinging at the wall junctions, i.e., at the nodes, could occur for two mechanisms^41^: the shearing of the cell wall material [$K_{{{\text{hg}}_{{{\text{global}}}} }}$, Eq. (2)] or the local bending of the wall [$K_{{{\text{hg}}_{{{\text{local}}}} }}$, Eq. (3)].

The values of the geometrical parameters are assigned as ranges by also considering the technological feasibility in terms of the printability of the structure with the selected FFF machine and material (see the Methods Sectio﻿n). According to the reference formula used, the angle $\theta$ must be negative, $\theta \in \left[ { - 60^{ \circ } ,0^{ \circ } } \right]$, the lengths of the diagonal $l$ and vertical rib $h$ must be in a defined range where $h,l \in \left[ {4{\text{ mm}},12{\text{ mm}}} \right]$, the thickness $t$ should be a multiple of the printing nozzle diameter, $t = \left[ {0.8{\text{ mm}},1.2{\text{ mm}},1.6{\text{ mm}}, \ldots } \right]$. Rib lengths should be at least five times as large as the rib thickness satisfying $5 - h/t \le 0$ and $5 - l/t \le 0$. The middle vertices of the unit cell must not overlap according to the following $1 - h - 2l\sin \theta + t/\cos \theta \le 0$. Finally, as in^41^, $q$ can be set equal to $l/10$.

**Figure 4:**
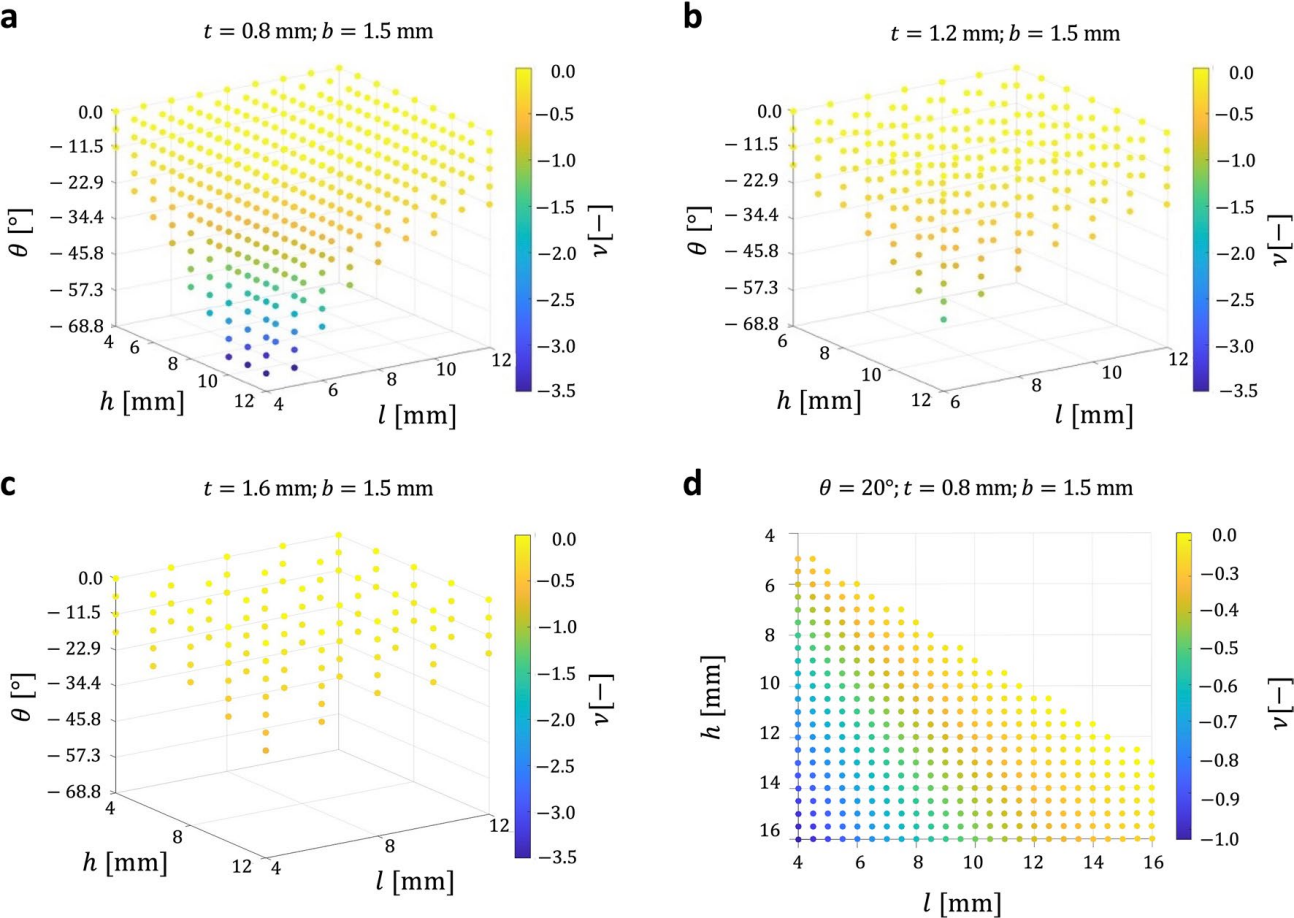
Scatterplots used to analyze the effect of the geometrical parameters $\theta$, $h,$ and $l$ on the Poisson's ratio $\nu$, based on the analytical equation [Eq. (1), considering $K_{{{\text{hg}}_{{{\text{global}}}} }}$ as hinging force constant], for fixed values of the $t$ and $b$ parameters, i.e., $t = 0.8 {\text{mm}}$ and $b = 1.5 {\text{mm}}$ (**a**), $t = 1.2 {\text{mm}}$ and $b = 1.5 {\text{mm}}$ (**b**), $t = 1.6 {\text{mm}}$ and $b = 1.5 {\text{mm}}$ (**c**), and for fixed values of $\theta$, $t$ and $b$ parameters (**d**, $\theta = 20^\circ ; t = 0.8 {\text{mm}};b = 1.5 {\text{mm}}$).

**Figure 5:**
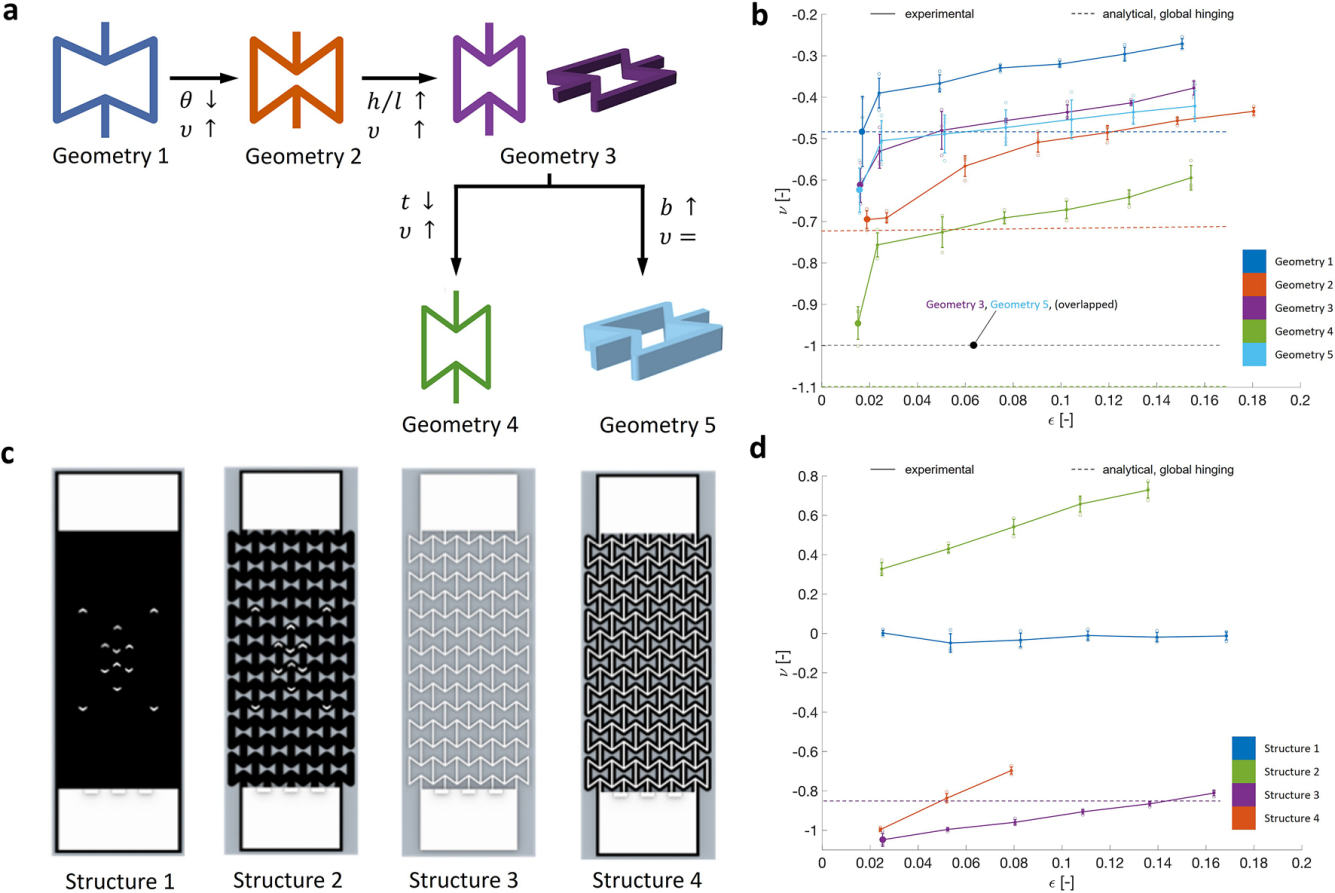
Experimental results (**a**) The five different geometries of reentrant honeycombs investigated. (**b**) Experimental results of the Poisson's ratio $\nu$ over the engineering strain $\varepsilon$ for the five geometries. In this chart also the Poisson’s value obtained from the analytical equations (global hinging) are mapped as lines (they do not depend on the engineering strain) (**c**) The four structures tested to measure the Poisson's ratio, involving Structure 1 as a standard KT, Structure 2 realized as standard KT with auxetic perforations, Structure 3 as a 3D-printed auxetic structure, and Structure 4 as a combination of Structures 2 and 3 glued to each other. Structures 1 and 2 include *v*-shaped marks as relevant points for measuring the Poisson’s ratio (**d**) Results of the Poisson's ratio $\nu$ over the engineering strain $\varepsilon$ for the four analyzed structures. In this chart, for Structure 3, also the Poisson’s value obtained from the analytical equations (global hinging) is mapped as a line.

The smallest Poisson's ratio is found by graphically analyzing Fig. 4a–c containing the scatterplots of the Poisson's ratio as a function of the geometrical parameters $\theta$, $h,$ and $l$ for fixed values of the parameters $t$ and $b$. They show that the smallest Poisson's ratio is achieved by minimizing $\theta$, maximizing $h$, and minimizing $l$. By comparing the scatterplots for different values of $t$, it can be noticed that a smaller $t$ results in a more negative Poisson's ratio; moreover, as $t$ increases, the scatter plot contains fewer points as smaller ranges of $\theta$, $h,$ and $l$ fulfill the constraints. Figure 4d shows that the Poisson's ratio remains approximately constant for a particular $h/l$ ratio, while increasing in magnitude as the ratio becomes larger. Based on the previous considerations, five geometries of the auxetic structure are investigated (Table 1 and Fig. 5a) for the sensitivity of the Poisson's ratio to each geometrical parameter. Tensile tests are conducted on 3D-printed five-by-five auxetic structures. The corresponding Poisson’s ratio of the structures is derived and calculated through image-based analysis (for further details, see the Methods Section). The values of the force constants and the Poisson's ratio computed analytically are reported in Table 1, using the material properties of the TPU ($E_{{\text{s}}}$ = 26 MPa^42^, $v_{{\text{s}}}$ = 0.48^43^). In Fig. 5b, we report the Poisson's ratio values of the five studied configurations as a function of the engineering strain. In this graph, the analytical values obtained, considering the $K_{{{\text{hg}}_{{{\text{global}}}} }}$ force constant in Eq. (1), are also mapped since those are closer to the experimental values. These values do not depend on the engineering strain. Hence, they are represented as a line.

**Table 1:** Geometrical parameters, force constants, and values of the Poisson's ratio $\nu$ from the analytical equation and experimental tests for the five geometries of reentrant honeycombs investigated according to Fig. 5a.

Geometry	Geometrical parameters					Force constants				Poisson's ratio $v$		
	*Θ*	*h*	*l*	*t*	*b*	$K_{{{\text{hg}}_{{{\text{global}}}} }}$	$K_{{{\text{hg}}_{{{\text{local}}}} }}$	$K_{{\text{f}}}$	$K_{{\text{s}}}$	Analytical		Experimental lowest strain
	[°]	[mm]				[MPa]				Global hinging	Local hinging	
1	− 24	12	8	1.2	1.5	1.98	0.22	0.13	5.85	− 0.48	− 0.50	− 0.48
2	− 36	12	8	1.2	1.5	1.98	0.22	0.13	5.85	− 0.72	− 0.75	− 0.70
3	− 36	12	6	1.2	1.5	2.64	0.52	0.31	7.80	− 0.99	− 1.06	− 0.61
4	− 36	12	6	0.8	1.5	1.76	0.15	0.09	5.20	− 1.12	− 1.17	− 0.94
5	− 36	12	6	1.2	3	5.27	1.04	0.62	15.60	− 0.99	− 1.06	− 0.62

The Poisson's ratio of all geometries becomes less negative with increasing engineering strain. The comparisons between the curves show that decreasing $\theta$ (from Geometry 1 to Geometry 2) or decreasing $t$ (from Geometry 3 to Geometry 4) results in a downward shift of the curve, increasing the magnitude of the Poisson's ratio. Oppositely, by increasing the $h/l$ ratio (from Geometry 2 to Geometry 3), the curve shifts upward, not matching the assumption from the analytical equation. This result occurs because by modifying the $h/l$ ratio, $l$ is decreased, but $t$ is kept constant (Fig. 3). Hence, the $t/l$ ratio increases, thus affecting the slenderness of the sloped walls of the cell which decreases. This change could affect the validity of the beam-based assumptions related to the analytical model. On the contrary, the slenderness of the vertical walls is kept constant because the $h$ value does not change. When these ratios are preserved as in Geometry 1 and Geometry 2 there is almost a perfect match between the analytical and experimental values (Table 1). Indeed, Geometry 4 has an increase in the Poisson’s ratio compared to Geometry 3 because, in this case, the slenderness of both the vertical and sloped beams decreases (we have reduced $t$, Table 1).

Theoretically, the prevalent deformation mechanism is the flexing (i.e., $K_{{\text{f}}}$) of the cell beams in all the studied geometries (i.e., it is the lowest *K* value which predominates^41^). However, the hinging at the nodes can become relevant when the length of the diagonal rib is reduced and $t$ is kept constant. Besides, the change of the $l$ value also affects the $q$ value (as in^41^, it was set equal to $l/10$) in the $K_{{{\text{hg}}_{{{\text{local}}}} }}$ [Eq. (3)]. It is also worth underlying that the shear modulus of the material in the hinge [Eq. (2)] is likely to be lower than the *G*_﻿S_ of the bulk material due to local damage^41^. Finally, the change of parameter *b* (from Geometry 3 to 5) does not influence the Poisson's ratio. This result is confirmed both analytically and experimentally (see Table 1). Indeed, all force constants are directly proportional to *b*. Hence, the parameter *b* cancels out in Eq. (1).

The experimental results show that the Poisson's ratio depends on the engineering strain and becomes less negative with increased strain. However, the analytical equation is independent of the engineering strain (they are represented as lines in Fig. 5b). The values obtained from the analytical equation represent the values from the experimental measurements at the lowest engineering strains (Table 1). Therefore, the analytical equation is only valid at small deformations. At larger deformations, the Poisson's ratio is dependent on the level of strain, as already underlined by Wan et al.^44^. The error is more significant for Geometry 3, 4, and 5. This is most likely due to the ratio $l/t$ not being sufficiently large. However, when comparing these three geometries to each other, the general assumptions from the analytical equation still hold.

Even though the analytical equation only provides a proper representation of the Poisson's ratio at low engineering strains, it proves to be a good indicator, in general, to study whether specific geometrical parameters enlarge or reduce the auxetic effect. It is a simple procedure to determine how sensitive the auxetic effect is to the influence of the geometrical parameters.

### Comparison between the standard and the auxetic KT

From the results obtained from the experimental measurements (Fig. 5b), the following unit-cell dimensions are chosen: *θ* = -36°; *h* = 12 mm; *l* = 6.5 mm; *t*_TPU_ = 0.8 mm; *b* = 1.5 mm; $t_{{{\text{TAPE}}}} = 3.5$ mm (Table 1). The unit-cell dimensions almost entirely equate the dimensions of Geometry 4, except for the length $l$ which is slightly increased to be able to design a perforated KT such that it covers a large enough area of patient’s skin to secure a proper adhesion. The value 6.5 mm is derived from applying the constraint which ensures that the middle corners of the unit cell do not overlap with the tape. The selected geometry both maximizes the auxetic effect and, at the same time, controls the out-of-plane deformation upon stretching of the overall auxetic structure.

A second experimental campaign is then conducted to evaluate the differences in Poisson's ratios of the standard and the auxetic KT based on the selected unit-cell geometry. Thus, four structures are investigated (Fig. 5c). The first consists of the standard KT. The second is fabricated by laser cutting a standard KT to create an auxetic perforation. In this case, we select $t_{{{\text{TAPE}}}} = 3.5$ mm where $t_{{{\text{TAPE}}}}$ corresponds to the rib thickness $t$ value of the unit-cell geometry. The $t_{{{\text{TAPE}}}} = 3.5$ mm value is set higher than $t_{{{\text{TPU}}}}$ so that the tape covers a larger area of the patient's skin for proper adhesion. The third structure (Fig. 5c) is a 3D-printed auxetic structure, while the fourth structure is the combination of the second and third structures comprising a laser-cut KT with a TPU auxetic structure 3D-printed on top of it (Fig. 5c). Figure 5d reports the results of tensile tests showing Poisson's ratio over the engineering strain of these four structures. Structure 1 exhibits a Poisson's ratio, which is close to 0 and independent of the strain, in accordance with the literature and manufacturers' indications related to standard KT. Structure 2 has a positive Poisson's ratio, meaning that the laser-cut KT by itself does not show any auxetic behavior. Structure 4 shows a Poisson's ratio that is less negative than Structure 3, as the tape realized as Structure 2 does not contribute to the auxetic behavior, in fact it does the opposite. The Poisson’s value obtained from the analytical equations (global hinging) is mapped as a line for Structure 3. As explained in the Methods Section, in the case of Structure 4 some values are not provided in this chart because non-planar deformations occurred at those points.

### Analysis of the possible application scenarios of the auxetic KT

**Figure 6:**
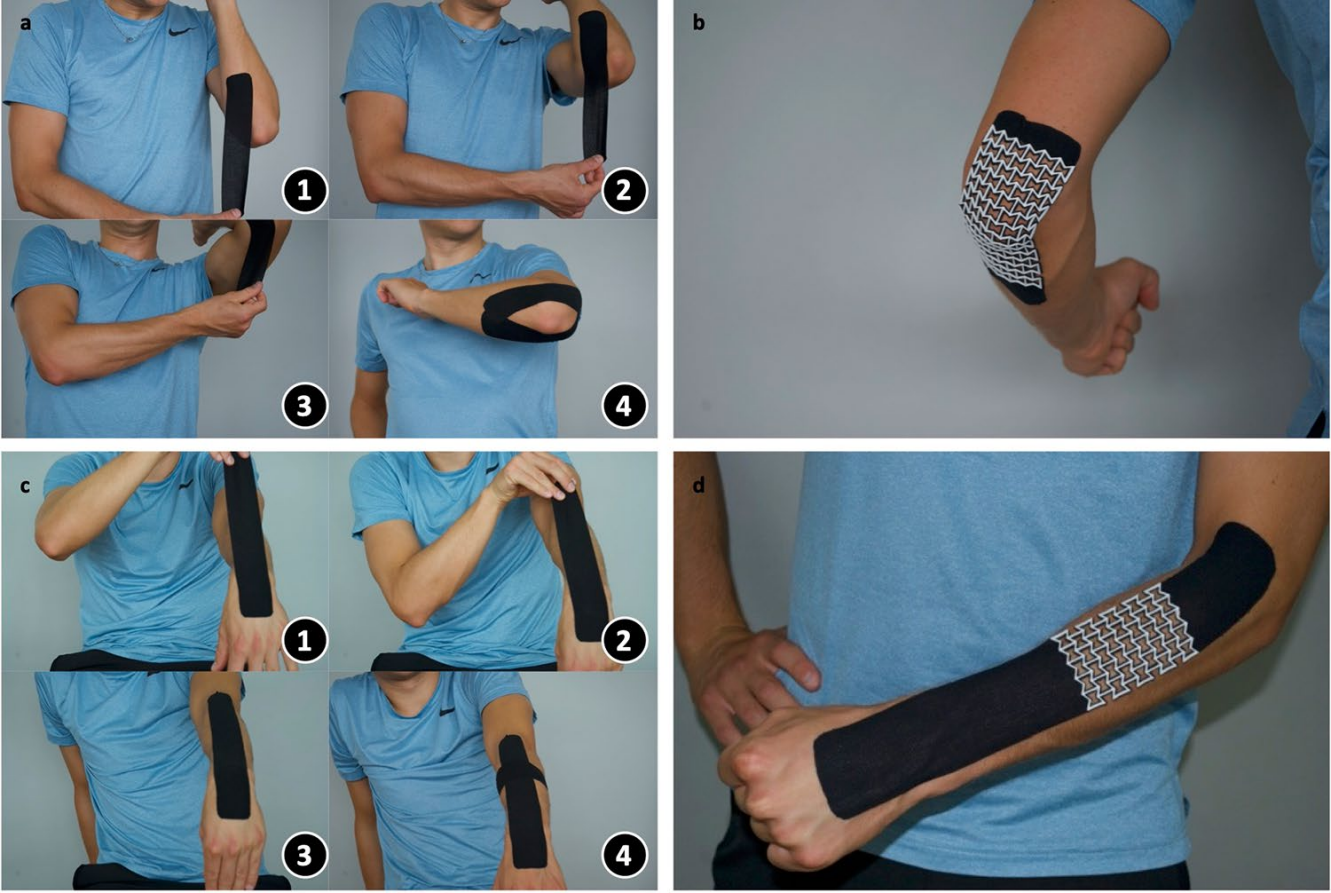
Comparison between the application procedure of the traditional KT and the novel auxetic KT for two cases. (**a**) The traditional KT application procedure for general elbow pain divided into 4 steps. (**b**) The auxetic KT for elbow pain. (**c**) The traditional KT application procedure for forearm muscle tenseness divided into 4 steps. (**d**) The auxetic KT for forearm muscle tenseness.

Although the application of KT covers many types of injuries, among them, two different but frequent application scenarios are chosen to explore the potential of the novel auxetic KT. In the first case, the auxetic KT is designed to treat general pain in the elbow due to overuse or overstretching of the elbow. Often, the patient experiences dull pain, which is not explicitly located in one area and can result in weakness of the joint. Therefore, a taping method for general elbow pain, which provides support and relieves pain by lifting the skin so that the patient can continue with activities, proves helpful. When using the traditional KT (Fig. 6a), the elbow is flexed, and one end of the first piece of tape is attached below the elbow with no tension. It is then applied along the medial side of the elbow with a stretch of 50% of the maximum possible tension of the tape. The second end is attached above the elbow with no stretch. The same procedure is repeated with another piece of tape along the lateral side of the elbow. When an auxetic structure is incorporated into the KT, the synclastic curvature will allow the tape to conform to the shape of the elbow (Fig. 6b). This effect permits the use of only one piece of auxetic tape instead of several parts. This leads to a simplification of the application procedure.

The second case is the application of the auxetic KT on the muscles of the forearm to treat muscle tenseness. This pain is caused by overuse or inflammation of the muscle, which creates a point of tension within the muscle. With the traditional KT (Fig. 6c), the muscle should be stretched by flexing the wrist into a downward position^21^. The first end of the tape is applied to the hand with zero tension. The tape is then applied along the length of the arm with a stretch of 25–50% of the maximum possible tension. No tension is applied on the second end of the tape. The stretching of the tape causes a convolution in the skin to reduce the pressure and relieve the pain. At the location of pain, a second shorter piece of tape is applied perpendicular to the first piece of tape, which aids in lifting the skin even more (Fig. 6c, Step 4). Using an auxetic structure at the intersection allows combining two pieces of tape into one; therefore, it simplifies the application procedure (Fig. 6d).

Based on these two applications, the following considerations were derived. In the case of the general elbow pain (Fig. 6a, b), the location of the auxetic KT compared to the standard KT is different. With the existing KT, the two pieces are placed around the elbow joint (Fig. 6a). Instead, the auxetic KT is placed directly over the joint (Fig. 6b). In this case, the joint can move freely, as the property of synclastic curvature allows a good adaptation of the tape to the shape of the joint and ensures unrestricted movements. An end-user could intuitively think that the auxetic tape restricts movements, as it covers the entire joint. However, when the tape is applied instead, the elbow can be moved freely (Fig. 6b). This proves that the synclastic curvature of the auxetic tape adds benefits to a KT.

Besides lifting the skin, the auxetic tape can also benefit the fascia (Fig. 1a, b). The fascia is the connective tissue underneath the skin, which encloses the muscles. Compared to the muscles, the fascia can cover a larger area. A standard KT, in most cases, is applied along the length of the muscles, and the stimulus on the muscle is one-dimensional. However, the auxetic KT can cover the larger area of the fascia. Therefore, the expansion of the auxetic tape in two directions is also valid for treating the fascia. The auxetic KT can thus support the fascia as it increases its tension. Furthermore, errors within the application procedure are reduced when using the auxetic KT to treat the fascia. These errors can occur when larger areas, such as the shoulder, are treated. Existing KT cannot cover such larger areas. Several longitudinal tapes would be needed, but the amount of tension in each tape should be the same. If this is not the case, one tape takes away the function of the other, leading to incorrect treatment. This issue can be avoided when using only one auxetic tape to cover the whole area.

Regarding the fabrication of the auxetic KT, an easy application requires that similarly to the existing KT, it is fabricated as one long strip and delivered on a roll. A physiotherapist or an athlete could thus easily cut the required length of tape from the roll. The current auxetic prototypes were fabricated using a 3D printing technology that does not allow the manufacturing of such rolls. Further fabrication techniques could be explored, such as the manufacturing of auxetic fabrics or auxetic yarn^45,46^. On one side of the auxetic fabric, an acrylic adhesive should be applied to guarantee the proper attachment to the skin. Besides, the rigidity of the auxetic tape should be adequately tuned, by adjusting the structure stiffness or creating auxetic KT from auxetic fabric, woven or 3D-printed. However, the auxetic KT rigidity represents an advantage for its personalization. For example, an increased rigidity might be helpful when an athlete is in the acute phase of the injury, during which no sports activities are allowed.

Therefore, the tape mixes the original inelastic athletic tape, which restricts movements, and the elastic KT, which allows free movements. The athlete can freely move due to the synclastic curvature, but still has increased injury support thanks to the auxetic structure. By properly designing this structure, the novel tape could also provide different levels of treatment.

## Conclusions

A novel KT has been developed based on auxetic structures. A combination of laser cutting and 3D printing is used to fabricate the novel auxetic tape. The Poisson's ratio is investigated through analytical equations and experimental tests. The auxetic KT proves to reduce the steps of the application procedure because it generates a two-dimensional treatment, provides comfort by allowing a free range of motion due to the synclastic property of auxetics, allows tuning the tape rigidity to allow personalizing its effect to the end-user’s skin and type of injury.

The novel KT has been designed using the reentrant honeycomb topology as a reference. Due to its simplicity, it allowed to use FFF to 3D print the structure and to laser cut the shape into the tape easily. However, further topologies can be also explored, involving auxetic structures with chiral building-blocks^47^, superelliptic parameterizations^48^, or even auxetic fabrics^31^, and as such creating geometries with fewer sharp corners. In that way, the selection of the topology could also consider heterogenous design aspects such as comfort but also fabrication constraints.

## Methods

### Design and prototyping

The novel auxetic KT is composed of two parts; an auxetic laser-cut KT and a corresponding 3D-printed TPU auxetic structure.

**Figure 7:**
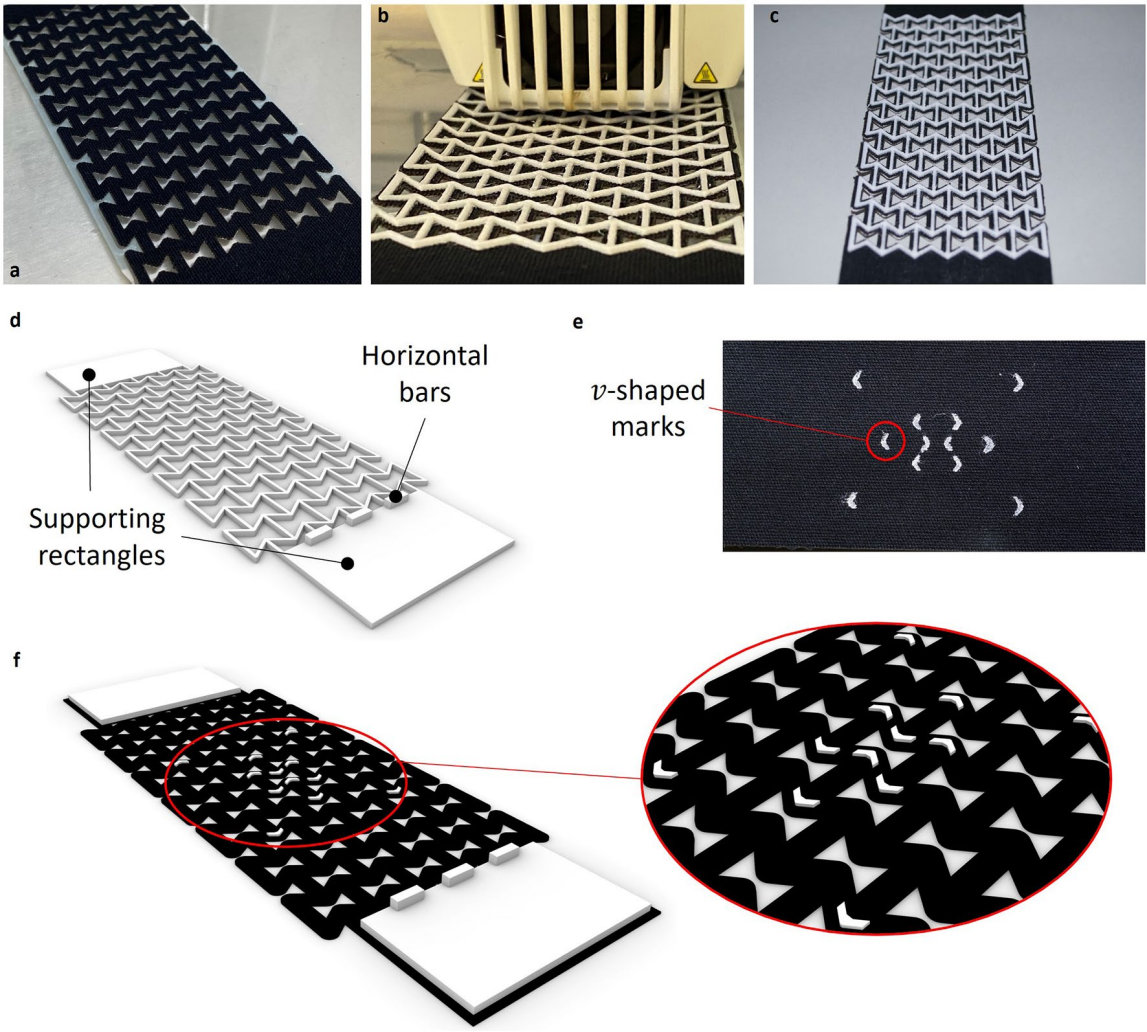
Fabrication steps of the auxetic KT. (**a**) The auxetic structure is laser cut into the KT. (**b**) The flexible material (TPU) is 3D-printed onto the tape. (**c**) Fabricated auxetic KT prototype. (**d**) Supporting rectangles and horizontal bars are added to the auxetic structures used for the mechanical characterization. (**e**) *v*-shaped marks indicated on the only tape samples (Structure 1 in Fig. 5c) as relevant points for measuring the Poisson’s ratio. (f) The sample used for Structure 2 (Fig. 5c) and a detail of the *v*-shaped marks.

First, the auxetic structure is fabricated by laser cutting (Model: Adcomm VREL Laser CO2-N 600/80, Software: RDWorksV8) a standard KT (Fig. 7a). Then, an auxetic structure built using the flexible TPU filament (Ultimaker TPU 95A^42^) is 3D-printed directly onto the tape (Fig. 7b, c) using FFF technology (Ultimaker S5, printing nozzle 0.4 mm diameter). The auxetic structures for the laser cutting and those for the 3D printing are modeled using Grasshopper 3D, which runs within the computer-aided design software Rhinoceros 3D (Version 7.4).

To prepare the 3D printing pre-processing files, the Ultimaker Cura slicer software (Version 4.8.0) is used. The standard printing settings for the Ultimaker TPU 95A filament are used with a few exceptions; the infill density of the filament is changed to 100%, whereas the z-offset is set to 0.5 mm, to roughly approximate the thickness of the tape. The tape needs to be placed precisely at the position on the printing bed, where the TPU filament will be printed, according to the Ultimaker Cura software. For all the four auxetic structures analyzed (Fig. 5c), supporting rectangles (Fig. 7d) are included in the design to position them in the tensile testing device and provide proper clamping during measurements. Along the edge of the bottom rectangle, three horizontal bars are added, ensuring that the structure is positioned straight (Fig. 7d). When comparing the existing and auxetic KT, *v*-shaped marks were drawn on Structure 1 and Structure 2 in Fig. 5c using a laser-cut mask (Fig. 7e, f) to properly define the points used for the Poisson’s ratio calculation through image-based analysis. Figure 7d also shows the type of samples that were 3D-printed and tested to obtain the Poisson’s ratio (Fig. 5b) of the five reentrant honeycomb topologies shown in Fig. 5a. In this case, the dimensions of the supporting rectangles vary with the auxetic topology.

### Mechanical characterization and data elaboration

**Figure 8:**
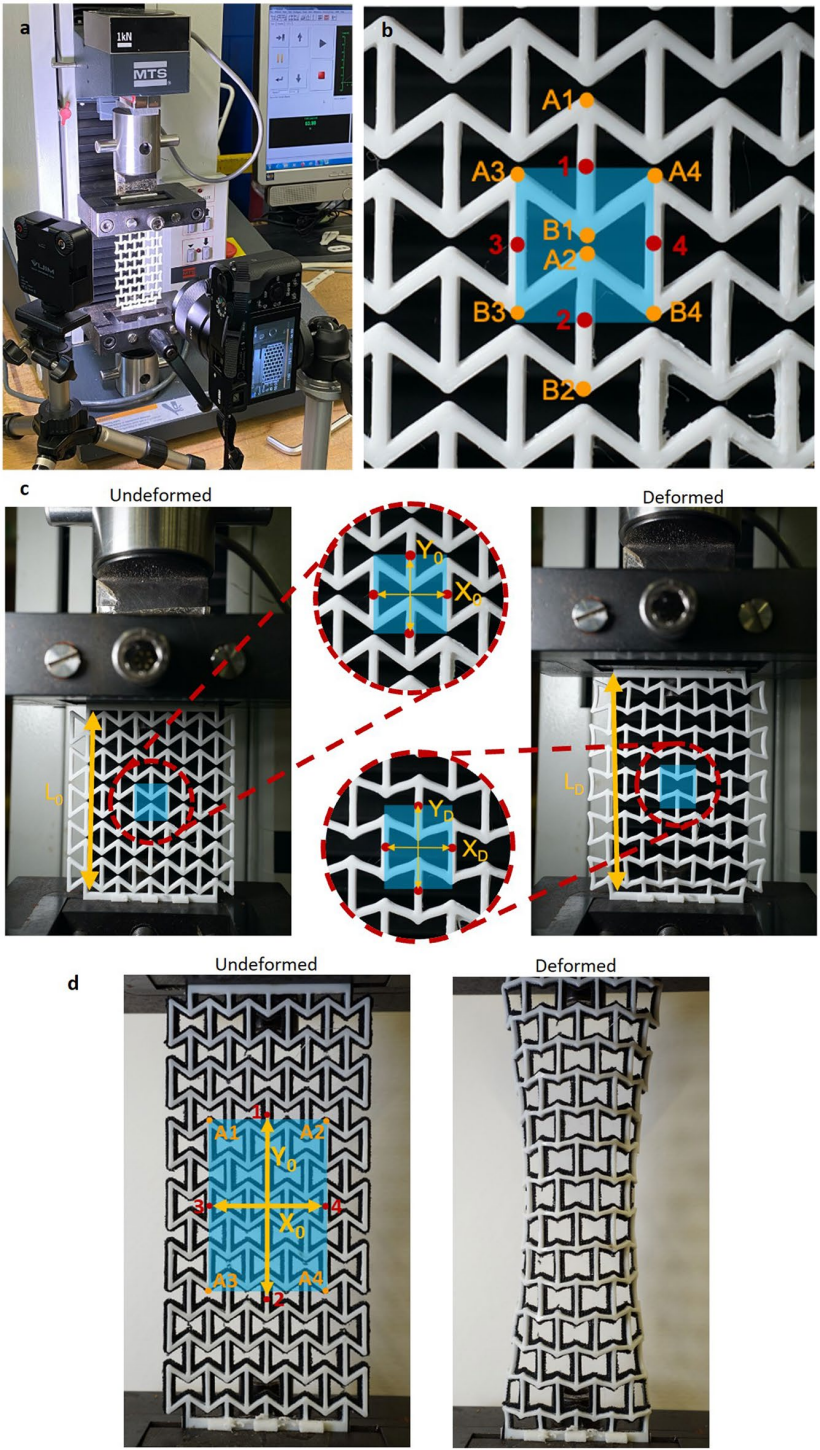
Mechanical characterization and data analysis. (**a**) Tensile test setup for the mechanical characterization. (**b**) Data analysis: the Poisson’s ratio is determined for the highlighted central unit cell. The coordinates of relevant points (labeled as A_*i*_ and B_*i*_, *i* = 1, 2, 3, 4) are computed in pixels, from which the coordinates of the middle point of each vertical beam (labeled simply using numbers from 1 to 4) are calculated. (**c**) Undeformed and deformed auxetic structure in the tensile test measurement device: $X_{0}$, $Y_{0}$, $X_{{\text{D}}}$ and $Y_{{\text{D}}}$ are used to calculate the Poisson’s ratio at the given strain, determined using the $L_{0}$ and $L_{{\text{D}}}$ measurements. (**d**) Undeformed and deformed auxetic KT. The points analyzed for this second experiment are also highlighted.

For the tuning of the auxetic structure design, tensile tests (Electromechanical Testing Systems, MTS Synergie 200, load cell 1kN) are performed on an auxetic structure containing five unit cells in both *x*- and *y*-direction. The test setup is shown in Fig. 8a. While the measurement device applies a tensile load with a speed of 5 mm/min on the structure, pictures are taken at different engineering strains with the Sony A6000 camera. The supporting rectangles of the structures (Fig. 7d) are clamped to the measurement device. The clamping of these parts restricts the auxetic behavior of the structure as it cannot expand in this area. Therefore, the Poisson’s ratio is determined for the central unit cell of the structure (highlighted in Fig. 8b), which is influenced the least by this end effect. Each picture (resolution of 350 × 350 dpi, containing 4000 × 6000 pixels), taken at specific engineering strains, is compared to the original undeformed image to calculate the Poisson’s ratio of the unit cell. There is no need to stop the testing machine at different strains to take pictures. The experiment runs continuously until deformation of 20 mm is reached. The camera is programmed to take a photo every 9 s for 30 shots in total. These images are analyzed in Matlab (Version Matlab R2020b). Four relevant points (labeled simply using numbers from 1 to 4 in Fig. 8b) are determined in the middle of the vertical ribs of the unit cell. Since they cannot be marked directly by hand, the corner points (A_*i*_ and B_*i*_, *i* = 1, 2, 3, 4 shown in Fig. 8b) are marked, and their coordinates are calculated in pixels. Hence, the coordinates of points labeled using numbers from 1 to 4 in Fig. 8b can be calculated. From these four points, the distances $X_{0}$ and $Y_{0}$ can be computed for the undeformed structure, and $X_{{\text{D}}}$ and $Y_{{\text{D}}}$ for the deformed one (Fig. 8c). Finally, the Poisson’s ratio is determined as $\nu = - \frac{{\varepsilon_{x} }}{{\varepsilon_{y} }} = - \frac{{\left( {X_{{\text{D}}} - X_{0} } \right)/X_{0} }}{{\left( {Y_{{\text{D}}} - Y_{0} } \right)/Y_{0} }}$. This calculation is done for the image taken at each strain (calculated using the $L_{0}$ and $L_{{\text{D}}}$ measurements shown in Fig. 8c as $\varepsilon = \frac{{L_{{\text{D}}} - L_{0} }}{{L_{0} }}$) to plot the Poisson’s ratio over the engineering strain. For each auxetic structure, three samples are tested. The average Poisson’s ratio is calculated for the central unit cell of the structure and plotted over the engineering strain. In addition, at each strain, the standard deviation of the three samples is calculated to evaluate the variance and consequently the repeatability.

For the second experiment related to the comparison between the standard and the auxetic KT, we design a five-by-seven structure. We use this higher number of cells to have a longer tape and thus reduce the influence of the clamping, especially in the case of the tape-only structure (i.e., Structure 1 in Fig. 5c). In this case, we do not analyze the Poisson's ratio of the central unit cell, but the Poisson's ratio of a larger area (Fig. 8d). This choice is made because this area can represent the possible extent of the patient's injury whose Poisson’s ratio is what matters for the application. Besides, it is also small enough to decrease the influence of the clamping.

Figure 8d, right, also shows the reason why fewer points are plotted in the chart in Fig. 5d for Structure 4. At a certain point a non-planar deformation occurs, and the calculation of the Poisson’s value is no longer valid. The five-by-seven structures are tested at 7 mm/min until a deformation of 28 mm is reached. The setup for the camera is the same as in the previous experiment.

## Data Availability

The datasets generated during and/or analyzed during the current study are available from the corresponding author on reasonable request.
